# Draft Genome Sequence of Ezakiella peruensis Strain M6.X2, a Human Gut Gram-Positive Anaerobic Coccus

**DOI:** 10.1128/genomeA.01487-17

**Published:** 2018-03-01

**Authors:** Awa Diop, Khoudia Diop, Enora Tomei, Didier Raoult, Florence Fenollar, Pierre-Edouard Fournier

**Affiliations:** a Unité de Recherche sur les Maladies Infectieuses et Tropicales Emergentes, Aix-Marseille Université, UM 63, CNRS UMR7278, IRD 198, INSERM U1095, Assistance Publique-Hôpitaux de Marseille, Institut Hospitalo-Universitaire Méditerranée-Infection, Faculté de Médecine, Marseille, France; b Special Infectious Agents Unit, King Fahd Medical Research Center, King Abdulaziz University, Jeddah, Saudi Arabia

## Abstract

We report here the draft genome sequence of Ezakiella peruensis strain M6.X2^T^. The draft genome is 1,672,788 bp long and harbors 1,589 predicted protein-encoding genes, including 26 antibiotic resistance genes with 1 gene encoding vancomycin resistance. The genome also exhibits 1 clustered regularly interspaced short palindromic repeat region and 333 genes acquired by horizontal gene transfer.

## GENOME ANNOUNCEMENT

Ezakiella peruensis is the type and only species of the genus Ezakiella, created in 2015 (1). E. peruensis occupies a unique position in an undefined family within the phylum Firmicutes (1). This microorganism is a Gram-positive anaerobic coccus. Gram-positive anaerobic cocci include many commensal species of humans and animals and also some human pathogens (2). The type strain M6.X2^T^ was isolated from a fecal sample of a healthy individual residing in a coastal traditional community in Peru (1). It is nonmotile and non-spore forming. Here, we present the annotated draft genome sequence of E. peruensis strain M6.X2^T^ (DSM 27367 = NBRC 109957 = CCUG 64571), which we obtained from the DSMZ collection.

Genomic DNA of E. peruensis strain M6.X2^T^ was sequenced using a MiSeq sequencer with the mate-pair strategy (Illumina, Inc., San Diego, CA, USA). DNA was quantiﬁed by a Qubit assay with a high-sensitivity kit (Life Technologies, Carlsbad, CA, USA) at 38.4 ng/µl. The 576,285 high-quality paired-end reads were trimmed and then assembled using the SPAdes assembler program (3). The draft genome sequence was annotated using Prokka software (4). Functional annotation was achieved using the BLASTp algorithm (5) against the Clusters of Orthologous Groups (COGs) database and the Rapid Annotations using Subsystems Technology (RAST) web server (6). Ribosomal RNAs (5S, 16S, and 23S rRNAs) were predicted using RNAmmer software (7).

The genome was 1,672,788-bp long, assembled in five scaffolds (seven contigs) with a G+C content of 36.9%. Overall, 1,589 protein-coding sequences were identified, including 1,165 (73.31%) protein-coding genes that had orthologs in the COGs database, 1,052 of which were assigned a putative function. A total of 46 tRNA loci and 1 rRNA operon (16S, 5S, and 23S rRNA) were identified in the genome. Strain M6.X2^T^ exhibited 26 genes associated with antibiotic resistance and toxic compounds, including one *vanW* gene encoding vancomycin resistance. No toxin/antitoxin module or bacteriocin-associated gene was identified. The genome of E. peruensis harbored 1 clustered regularly interspaced short palindromic repeat locus of 763 bp with 12 repeats (mean repeat length = 36 bp). We also detected 333 putative genes acquired by horizontal gene transfer, including 209 from bacteria within the order Clostridiales.

### Accession number(s).

The 16S rRNA and genome sequences from Ezakiella peruensis strain M6.X2^T^ are available in GenBank under accession numbers KJ469554 and OCSL00000000, respectively.
